# Characterization of the sexual reproductive process in the perennial tree *Robinia pseudoacacia*

**DOI:** 10.48130/forres-0026-0027

**Published:** 2026-09-08

**Authors:** Hong Lin, Xiao-Jing Wang, Kuan Sun, Chun-Lin Yan, Kai-Xun Guan, Ting Feng, Jie Yang, Keming Luo, Changzheng Xu, Jiang-Guo Meng

**Affiliations:** 1 School of Life Sciences, Southwest University, Chongqing 400715, China; 2 Key Laboratory of Eco-environments of Three Gorges Reservoir Region, Ministry of Education, School of Life Sciences, Southwest University, Chongqing 400715, China; 3 Nanshan Botanical Garden, Chongqing 400065, China

**Keywords:** Fertilization, Perennial plants, Pollen, *Robinia pseudoacacia*, Sexual reproduction

## Abstract

Sexual reproduction in perennial plants, especially woody species, remains poorly understood, and most studies have focused on annuals such as *Arabidopsis thaliana*. This gap hinders understanding of perennial reproductive processes and limits tree breeding. Here we characterized the sexual reproductive process of *Robinia pseudoacacia*, a perennial leguminous tree, and established standardized methods for studying its reproduction. We optimized cytological assays for pollen viability, *in vitro* germination, and pollen tube dynamics, achieving over 90% pollen viability and germination efficiency within 8 h. *R. pseudoacacia* pollen exhibited reproductive thermotolerance and maintained over 20% germination at 37 °C, a temperature lethal to *A. thaliana* pollen. This thermotolerance was associated with maintenance of stable plasma membrane integrity and an organized F-actin cytoskeleton. *R. pseudoacacia* pollen also germinated on *A. thaliana* stigmas, penetrated the styles, and targeted 3.6% of *A. thaliana* ovules, although post-zygotic incompatibility prevented seed formation. The ANX-RALF signaling module is conserved in *R. pseudoacacia*, and antisense oligodeoxynucleotide-mediated suppression of *RpANX2* disrupted pollen tube growth. Moreover, aberrant pollen tube guidance was linked to its low seed set. These findings provide a systematic characterization of sexual reproduction in a perennial tree and establish methodological foundations for reproductive studies in woody perennials, with implications for tree breeding.

## Introduction

Angiosperms dominate terrestrial ecosystems, accounting for over 90% of all land plant species^[1,2]^. Their diverse sexual reproductive mechanisms are a main driver of the exceptional biodiversity and ecological adaptability of this lineage^[3]^. Elucidating these mechanisms is critical not only for understanding plant adaptive strategies but also for advancing crop breeding^[4,5]^. However, reproductive research to date has focused almost exclusively on annual model species such as *Arabidopsis thaliana* (*A. thaliana*), *Oryza sativa*, and *Nicotiana alata*^[6−8]^. In these species, the cellular and molecular pathways governing reproductive organ development, fertilization, and embryogenesis have been extensively characterized, and the resulting knowledge has been successfully translated into crop breeding, for instance, in the development of male sterile lines that facilitate hybrid rice production^[9,10]^.

A substantial fraction of angiosperms, however, are perennials, whose reproductive strategies differ markedly from those of annual models. Many perennial forestry and fruit tree species possess substantial ecological and economic value, yet their reproductive biology remains poorly characterized. Flowering in these species may integrate cues accumulated over previous years, including environmental stresses such as drought and heat, as well as prior reproductive output. This integrative pattern is exemplified by the masting behavior of long-lived taxa^[11−13]^. After their initial flowering, adult perennials undergo repeated cycles of vegetative and reproductive growth, a process largely inaccessible to annual models^[14]^. Gamete development is similarly diverse, with female gametophyte morphology in perennials differing strikingly from *Arabidopsis*^[15]^. Although many perennials undergo conventional double fertilization, others reproduce by apomixis, a trait rare among annual models but of considerable significance for crop improvement^[16,17]^. In addition, some perennial taxa exhibit a notable capacity for interspecific and intergeneric hybridization, as demonstrated in *Catalpa*, *Malus*, and *Pyrus*, although the underlying cellular and molecular mechanisms remain poorly understood^[18,19]^.

Despite their scientific and practical importance, studies of perennial reproduction remain sparse^[20]^. Most are species-specific, yielding dispersed and poorly integrated datasets^[14,21]^. This fragmentation results largely from the absence of a unifying experimental system analogous to *Arabidopsis*. Ideally, such a system would combine tree-specific reproductive traits, a short juvenile phase, accessible floral organs, an extended flowering period, and genomic and genetic tractability. The woody systems currently available only partially meet these requirements. *Populus*, the best-established tree model, offers mature genetic transformation protocols and extensive genomic resources^[22]^, but its prolonged juvenile phase, inconspicuous wind-pollinated catkins, and narrow flowering window constrain reproductive experimentation. Other perennials, such as *Malus* and *Citrus*, have yielded valuable insights into specific processes, including hybridization and apomixis^[16−19]^, but their long generation times limit their utility as general systems. A mechanistic understanding of perennial reproduction therefore requires the systematic characterization of additional, experimentally tractable species.

*Robinia pseudoacacia* L. (hereafter referred to as *R. pseudoacacia*), commonly known as black locust, is a perennial leguminous tree native to North America and now naturalized across temperate and semi-arid regions worldwide^[23−25]^. As a pioneer species with an extensive root system, symbiotic nitrogen fixation, and high salinity tolerance, it rapidly colonizes degraded habitats and is widely used in ecological restoration^[25−29]^. It also provides nectar for apiculture, high-quality timber, and potentially protein-rich foliage for animal feed^[30,31]^. Beyond its practical and ecological values, its reproductive features, including abundant and accessible flowers, make *R. pseudoacacia* an interesting subject for studying sexual reproduction in perennials.

In this study, we present a comprehensive characterization of the reproductive process in *R. pseudoacacia*, encompassing events from pollen germination to the completion of fertilization. Through comparative analyses with *A. thaliana*, we identify reproductive features of *R. pseudoacacia* that are uncommon in annual models, including rapid pollen tube growth, exceptional thermotolerance, and the capacity to overcome intergeneric pre-fertilization barriers. We further develop cytological and methodological protocols for this species. These findings deepen our understanding of sexual reproduction in a woody perennial and lay the groundwork for future mechanistic and breeding studies.

## Materials and methods

### Plant materials

*A. thaliana* plants were grown at 22 °C under a 16 h light/8 h dark photoperiod. *R. pseudoacacia* L. flowers were collected from naturally growing trees in Nanshan Botanical Garden, Chongqing, China (29°31'9.95" N, 106°19'11.13" E), during the flowering season in April.

### Pollen tube germination assays

For semi-*in vitro* pollen germination, stigmas of *A. thaliana* and *R. pseudoacacia* were pollinated with fresh *R. pseudoacacia* pollen. After 15 min, the pollinated stigmas were excised, placed on solid pollen germination medium (PGM; 1.5 mM CaCl_2_, 1.5 mM Ca(NO_3_)_2_, 1 mM MgSO_4_, 0.01% H_3_BO_3_, 18.0% sucrose and 1.0% agarose, pH 7.0) and cultured for 3 h at 28 °C in the dark. For *in vitro* pollen tube growth, *R. pseudoacacia* pollen grains were germinated on solid PGM supplemented with 0.01 mg·L^−1^ stigma and style tissue exudates (STE) and cultured for 3 h at 28 °C in the dark.

### Pollen viability assays

*R. pseudoacacia* pollen grains were stained with 4',6-diamidino-2-phenylindole (DAPI; 10 μg·mL^−1^ for 10 min; Yeasen, cat. no. 40727ES10) or with Alexander stain (10 min; Coolaber, cat. no. SL7660). DAPI-stained pollen was observed under ultraviolet illumination, and Alexander-stained pollen was observed under bright-field, using a ZEISS Axioskop2 microscope.

### Pollen tube growth and thermotolerance assays

For pollen tube growth assays, *R. pseudoacacia* pollen grains were germinated in liquid PGM (1.5 mM CaCl_2_, 1.5 mM Ca(NO_3_)_2_, 1 mM MgSO_4_, 0.01% H_3_BO_3_ and 18.0% sucrose, pH 7.0) supplemented with Brefeldin A (BFA; 5 μg·mL^−1^) or Concanavalin A (ConcA; 1 μM). Pollen tube growth was recorded by time-lapse microscopy at 0, 10, and 20 min, and pollen tube length was measured over a 50-min time course.

For pollen germination thermotolerance assays, pollen grains were germinated on solid PGM at 22, 25, 28, 31, 34, or 37 °C, and germination rates were recorded after 8 h. For pollen tube thermotolerance assays, pollen grains were germinated on solid PGM at 28 °C for 3 h in the dark and were then subjected to heat shock by rapidly transferring the culture plates to a pre-equilibrated 37 °C incubator for 30 min. The pollen tubes were subsequently stained with FM4-64 (5 μM for 10 min; MedChemExpress, CAS 162112-35-8), gently washed twice with liquid PGM, and imaged. FM4-64 fluorescence was imaged using excitation at 561 nm and emission at 570–630 nm, and images were processed with Olympus cellSens software.

### Pollen tube guidance assays

For the pollen tube attraction assay, *R. pseudoacacia* or *A. thaliana* pollen grains were germinated *in vitro, *or pollen tubes were grown through excised stigmas by semi-*in vitro *culture. Unfertilized ovules were placed on both sides of a growing pollen tube tip with the micropyle oriented toward the pollen tube and were cultured for 3 h at 28 °C in the dark. Pollen tube guidance toward the ovules was observed under a light microscope.

For the AtLURE1.2 response assay, the mature AtLURE1.2 peptide (lacking the N-terminal signal peptide) was expressed in Escherichia coli as a His-tagged fusion protein and purified as described by Meng et al.^[32]^. The purified peptide was uniformly mixed into pollen germination medium to a final concentration of 1 μM. An equal volume of peptide buffer without the peptide was used in control assays. *R. pseudoacacia* and *A. thaliana* pollen grains were germinated on solid PGM at 28 °C for 3 h in the dark, treated with the peptide-containing medium for 1 h, and then observed and recorded by microscopy. A pollen tube was scored as responsive when its growth direction changed markedly during the treatment relative to the straight growth observed in controls. Twelve pollen tubes (*n* = 12) were examined per condition, and the assay was repeated at least three times.

### Aniline blue staining and microscopy

*A. thaliana* flowers at stage 12c were emasculated one day before pollination. Fresh *R. pseudoacacia* pollen grains were applied to mature *A. thaliana* or *R. pseudoacacia* stigmas. After 8 h, pistils were excised and fixed overnight in Carnoy's fixative (ethanol : acetic acid = 3:1, v/v). The fixed pistils were washed three times with 50 mM PBS buffer (Na_2_HPO_4_/NaH_2_PO_4_, pH 7.0), treated with 1 M NaOH for 5 h, washed again with 50 mM PBS buffer, and stained overnight with 0.1% aniline blue solution in 0.1 M K_3_PO_4_ (pH 8.0)^[33]^. The pistils were observed under ultraviolet illumination using a fluorescence microscope (ZEISS, Axioskop2).

### Confocal microscopy

Pollen grains were germinated on solid PGM for 3 h at 28 °C. The following fluorescent probes, each dissolved in liquid PGM, were applied separately to pollen tubes growing on the medium: 4,5-diaminofluorescein diacetate (DAF-DA; 10 μM for 10 min; Beyotime, cat. no. S0019S) for nitric oxide, 2',7'-dichlorodihydrofluorescein diacetate (H2DCFDA; 1 μM for 10 min; MedChemExpress, CAS 4091-99-0) for reactive oxygen species, Actin-Tracker Green-488 (1:50 dilution for 30 min; Beyotime, cat. no. C2201S) for F-actin, and FM4-64 (5 μM for 10 min; MedChemExpress, CAS 162112-35-8) for plasma membrane labeling. After incubation, the pollen tubes were washed twice with liquid PGM before imaging. Confocal images were acquired using an FV3000 laser scanning confocal microscope (Olympus). DAF-DA, H2DCFDA, and Actin-Tracker Green-488 signals were imaged using excitation at 488 nm and emission at 505–530 nm. FM4-64 signals were imaged using excitation at 561 nm and emission at 570–630 nm. Images were processed with Olympus cellSens software.

### AS-ODN selection and delivery to pollen tubes

Antisense oligodeoxynucleotides (AS-ODNs) were designed against the target gene using Beacon Designer, and 21-nucleotide antisense fragments were selected from the target mRNA. All ODNs were modified with phosphorothioate linkages at both the 5' and 3' ends, and an Alexa Fluor 488 moiety was conjugated to the 5' end to allow visualization of ODN uptake. Sense-strand ODNs (S-ODNs) of the same length and modifications served as controls. Two independent AS-ODN/S-ODN pairs (AS-*RpANX*2-1 and AS-*RpANX*2-2, with their corresponding sense controls S-RpANX2-1 and S-RpANX2-2) targeting distinct regions of the *RpANX2* transcript were used. ODN sequences are listed in Supplementary Table S1. All ODNs were synthesized by Zixi Biotechnology Co., Ltd (Qingke, China). For delivery, ODNs were first mixed with 10 μL of Trans Enhancer, followed by the addition of 0.3 μL of Rfect V2 transfection reagent (Baidai, China), and the mixture was incubated at room temperature for 10 min to allow the formation of ODN-cationic polymer complexes. The complexes were then diluted in liquid PGM to a final ODN concentration of 1 μM. Pollen grains were germinated on this medium and incubated for 3−6 h at 28 °C in a humidity chamber. ODN uptake by pollen tubes was verified by confocal microscopy (excitation, 488 nm; emission, 505−530 nm). Three independent experiments were performed.

### RT-PCR analysis

Total RNA was extracted from *R. pseudoacacia* pollen using the Bio-Spin Plant Total RNA Extraction Kit (Bioflux). First-strand cDNA was synthesized with the PrimeScript RT reagent Kit containing gDNA Eraser (Takara) following the manufacturer's recommendations. RT-PCR was performed with gene-specific primers. Two independent primer pairs (rt-*ANX2*-1F/R and rt-*ANX2*-2F/R) targeting distinct regions of the *RpANX2* transcript were used to cross-validate amplification specificity. *RpActin* served as the internal reference gene. All primer sequences are listed in Supplementary Table S1.

### Sequence and protein structure analyses

For sequence alignment, the complete protein sequences of *R. pseudoacacia* were retrieved from the NCBI protein database. Multiple sequence alignment was performed using TBtools^[34]^ to identify homologs of AtANX2, AtPRK6, AtRALF4, and AtRALF19 in *R. pseudoacacia*. Subsequently, the identified homologous sequences were imported into MEGA6 software for phylogenetic and homology analysis^[35]^, and the alignment results were visualized with Jalview. For structural analysis, the three-dimensional structures of the target proteins were predicted using AlphaFold2, and structural superposition was performed in PyMOL. Root mean square deviation (RMSD) values were calculated to assess structural similarity.

### Statistical analyses

All experiments involving imaging and quantification were repeated at least three times with similar results. Statistical analyses were conducted in GraphPad Prism 9.5. Two-tailed Student's *t-*tests were used to assess significant differences (* *p* < 0.05, ** *p* < 0.01, *** *p* < 0.001; N.S., not significant). Data are presented as mean ± SEM.

## Results

### Establishment of cytological methods for pollen biology in *R. pseudoacacia*

To develop a standardized cytological platform for *R. pseudoacacia*, we systematically optimized assays for pollen viability, *in vitro* germination, and pollen tube dynamics. For pollen viability assessment, mature pollen grains were simultaneously stained with DAPI (4',6-diamidino-2-phenylindole) and Alexander solution. Microscopic examination revealed intact DAPI-positive pollen nuclei and uniformly purple-stained cytoplasm, confirming the reliability of this dual-staining approach (Fig. 1a). Quantitative analysis further demonstrated an average pollen viability of over 90%, validating the high quality of *R. pseudoacacia* pollen (Fig. 1b).

**Figure 1:**
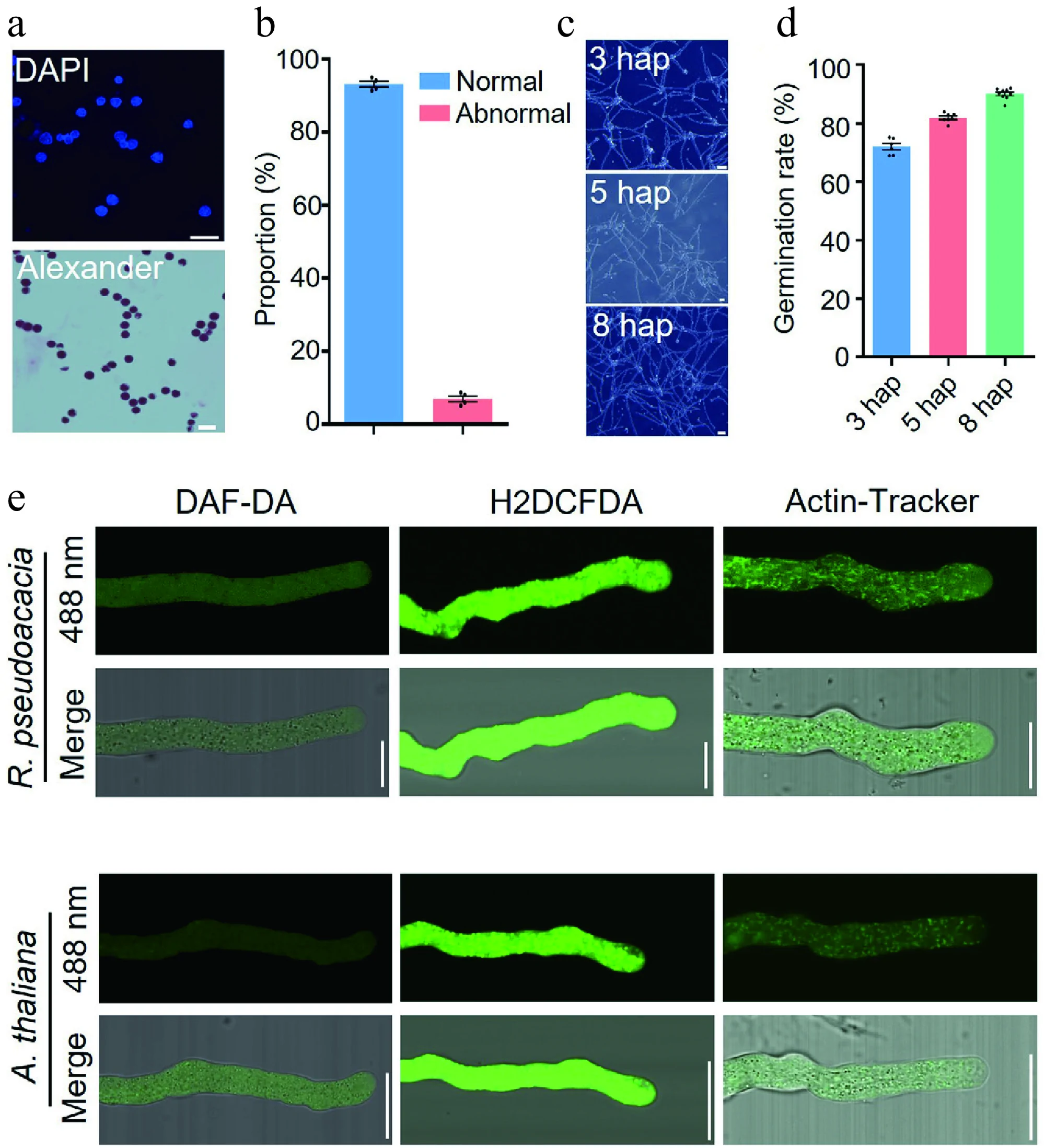
Establishment of cytological methods for studying *R. pseudoacacia* pollen tubes. (a) 4',6-Diamidino-2-phenylindole (DAPI) staining (upper panel) and Alexander staining (lower panel) of *R.* *pseudoacacia* pollen grains. DAPI labels nuclei (blue); Alexander staining distinguishes viable (darkly stained) from non-viable (pale) pollen grains. Scale bars, 20 μm. (b) Proportions of viable (Normal) and non-viable (Abnormal) pollen grains based on Alexander staining as shown in (a). Data are mean ± SEM (*n* = 4 biological replicates, ~200 grains per replicate); each dot represents one biological replicate. (c) *In vitro* germination of *R. pseudoacacia* pollen at 28 °C, imaged at 3, 5, and 8 hap. Scale bars, 20 μm. (d) Pollen germination rates at the time points shown in (c). Data are mean ± SEM (*n* = 6, 7, and 9 biological replicates for 3, 5, and 8 hap, respectively, ≥ 400 grains per replicate); each dot represents one biological replicate. (e) Confocal images of nitric oxide (NO), reactive oxygen species (ROS), and filamentous actin (F-actin) in pollen tubes of *R. pseudoacacia* and *A. thaliana*, visualized with 4,5-diaminofluorescein diacetate (DAF-DA), 2',7'-dichlorodihydrofluorescein diacetate (H2DCFDA), and Actin-Tracker, respectively. For each species, the upper row shows the fluorescence channel and the lower row shows the merged fluorescence-bright-field image. Scale bars, 10 μm.

To address previously reported low* in vitro *germination rates of *R. pseudoacacia* pollen^[36]^, we formulated a species-specific germination medium. Pollen tubes emerged as early as 3 hours after pollination (hap), followed by rapid elongation (Fig. 1c, d). By 8 hap, the germination rate exceeded 90%, marking the peak germination period under optimized conditions (Fig. 1c, d). Beyond 8 h, germination plateaued, with tube elongation becoming the predominant process. This high germination efficiency establishes the optimized medium as a platform for *R. pseudoacacia* pollen tube research.

Furthermore, we assessed the applicability of fluorescent probes routinely employed in *Arabidopsis* research to visualize key cellular components in *R. pseudoacacia* pollen tubes. DAF-DA enables real-time monitoring of nitric oxide (NO) dynamics, which is critical for pollen tube guidance and stress responses. H2DCFDA visualizes reactive oxygen species (ROS) production patterns that govern pollen tube polarity and membrane integrity. Actin-Tracker reveals F-actin cytoskeletal organization essential for vesicle trafficking and tip growth mechanics. These signaling molecules and cytoskeletal elements are fundamental to maintaining cellular polarity, particularly in highly polarized cells, where directional growth relies on their precise spatiotemporal coordination^[37,38]^. Confocal imaging revealed uniformly distributed NO and ROS, together with a dense actin fringe, in *R. pseudoacacia* pollen tubes, patterns that are indistinguishable from those observed in *A. thaliana* (Fig. 1e). These findings confirm that the established molecular probes from *A. thaliana* are compatible with *R.* *pseudoacacia*, establishing a framework for comparative reproductive studies. This compatibility will facilitate investigations into the signaling mechanisms underlying the distinctive reproductive traits of *R. pseudoacacia*.

### Growth dynamics and thermotolerance of *R. pseudoacacia* pollen

We first compared the growth kinetics of *R. pseudoacacia* pollen with those of *A. thaliana*. Under control conditions, *R. pseudoacacia* pollen tubes grew significantly faster (Fig. 2a). To test whether core growth mechanisms are conserved, we treated pollen tubes with the vesicle trafficking inhibitors Brefeldin A (BFA) and Concanavalin A (ConcA). BFA disrupts Golgi-mediated transport, and ConcA impairs membrane recycling. BFA almost completely inhibited pollen tube elongation, and ConcA severely disrupted tip growth polarity (Fig. 2a, b). These phenotypes closely resemble those observed in *A. thaliana*, suggesting that vesicle-mediated cytoskeletal dynamics are conserved in the pollen tubes of both species.

**Figure 2:**
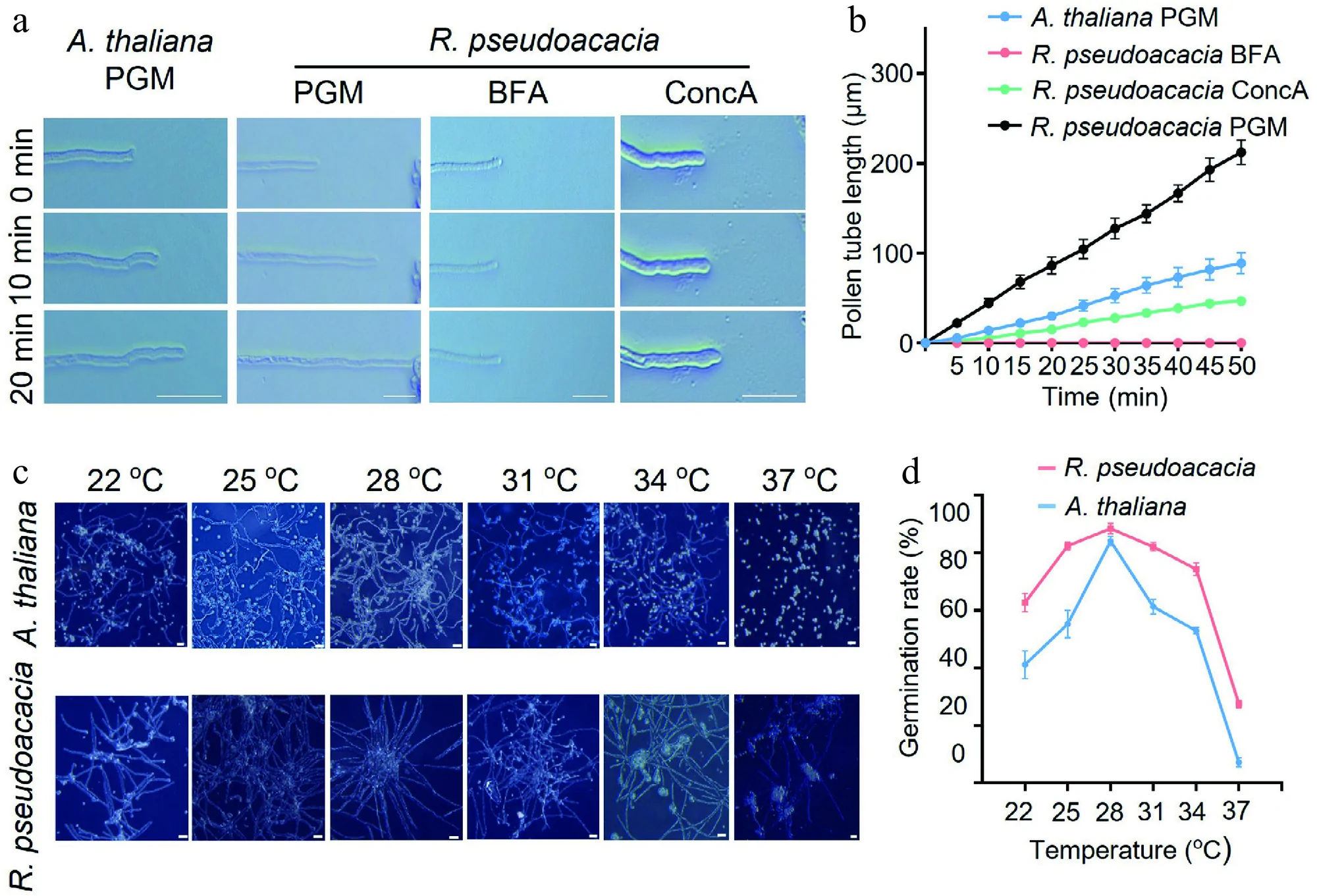
Growth dynamics and thermotolerance of *R. pseudoacacia* pollen. (a) Time-lapse images of *A. thaliana* and *R. pseudoacacia* pollen tubes growing in pollen germination medium (PGM) or in PGM supplemented with Brefeldin A (BFA) or Concanavalin A (ConcA), recorded at 0, 10, and 20 min. Scale bars, 20 μm. (b) Pollen tube length over a 50-min time course for the conditions shown in (a). Data are mean ± SEM. (c) Representative images of *A. thaliana* and *R. pseudoacacia* pollen germinated *in vitro* at temperatures ranging from 22 to 37 °C. Scale bars, 20 μm. (d) Pollen germination rates at each temperature are shown in (c). Data are mean ± SEM.

We next assessed pollen performance across a temperature gradient of 22 °C to 37 °C. *R. pseudoacacia* showed substantial thermotolerance. Germination efficiency exceeded 70% between 25 and 34 °C, and over 20% of pollen grains still germinated at 37 °C, a temperature lethal to *A. thaliana* pollen (Fig. 2c, d). By contrast, *A. thaliana* pollen failed to germinate at 37 °C (Fig. 2c, d). This heat resistance may contribute to the species' ability to colonize diverse climatic zones.

### Stable plasma membrane integrity and F-actin cytoskeleton underpin high-temperature tolerance

To investigate the cellular basis of thermotolerance in *R. pseudoacacia* pollen, we first examined plasma membrane integrity using the lipophilic dye FM4-64. At 28 °C, both *A. thaliana* and *R. pseudoacacia* pollen tubes displayed intact plasma membranes (Fig. 3a, c). Following heat shock at 37 °C (shifted from 28 °C), *A. thaliana* pollen tubes exhibited severe membrane damage. Only 21.2% retained membrane integrity, whereas the remainder showed tip vacuolation (15.4%), abnormal branching (28.8%), compromised membrane integrity (19.2%), or cell death (15.4%) (Fig. 3b). In contrast, 95.7% of *R. pseudoacacia* pollen tubes maintained normal membrane morphology at 37 °C, and only 4.3% displayed small vesicles budding from the plasma membrane (Fig. 3d). These results indicate that *R. pseudoacacia* possesses a mechanism for preserving plasma membrane stability under heat stress.

**Figure 3:**
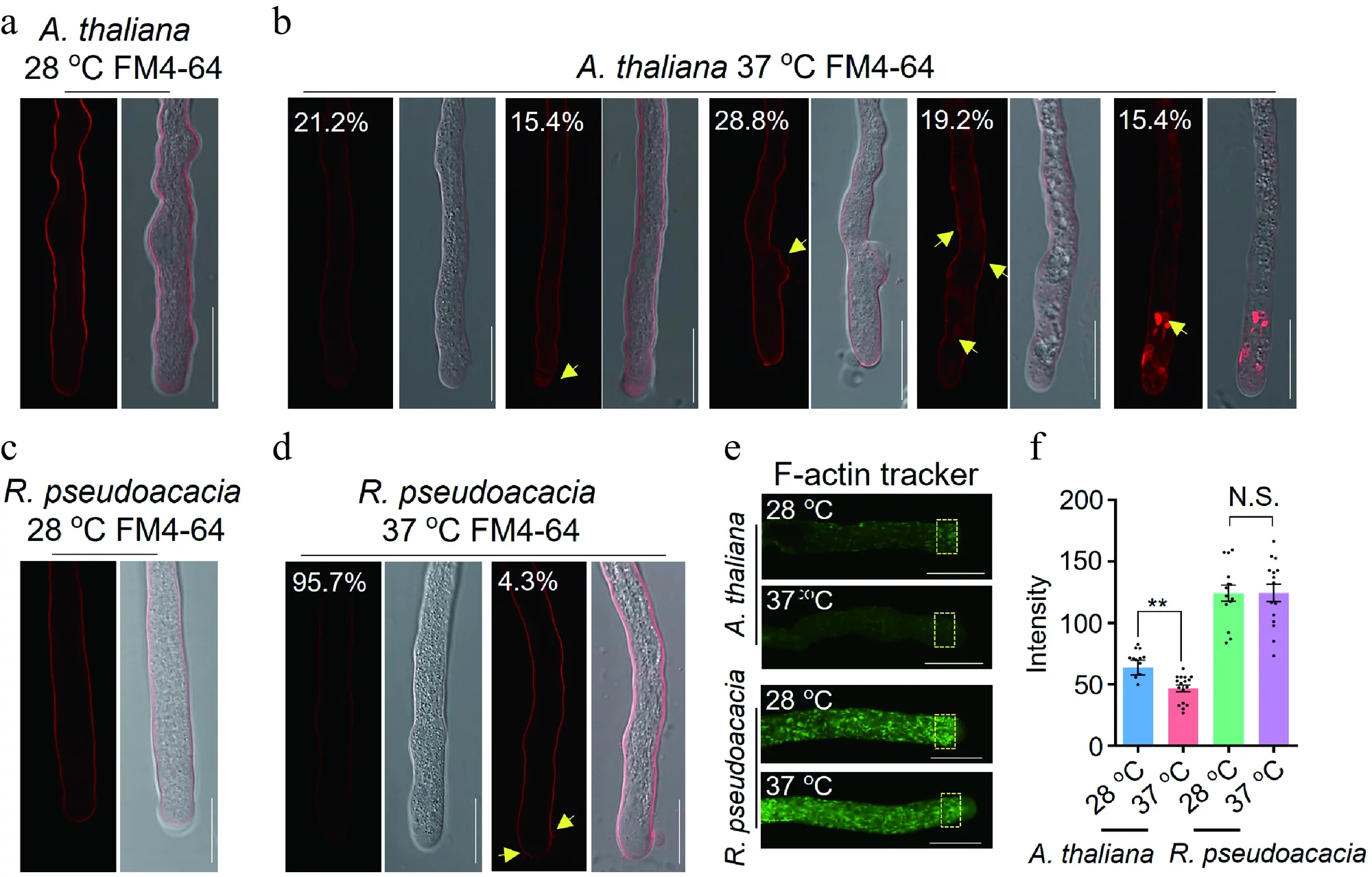
Stable plasma membrane integrity and F-actin cytoskeleton stability underlie high-temperature tolerance of *R. pseudoacacia* pollen tubes. (a) An *A. thaliana* pollen tube stained with the membrane-selective fluorescent dye FM4-64 after growth at 28 °C, showing intact plasma membranes. Left, fluorescence image; right, merged fluorescence-bright-field image. (b) Damage phenotypes in *A. thaliana* pollen tubes after heat shock (28 °C for 3 h in the dark, then 37 °C for 30 min), arranged from left to right in order of increasing severity: an intact plasma membrane (21.2% of tubes); vacuolation at the tube tip (15.4%); abnormal branching (28.8%, yellow arrowhead); intensified intracellular FM4-64 fluorescence accompanied by numerous large vesicles, indicating compromised plasma membrane integrity (19.2%, yellow arrowheads); and massive intracellular FM4-64 accumulation with bright-field evidence of cell death (15.4%, yellow arrowhead). Percentages indicate the proportion of each phenotypic class (*n* = 52 pollen tubes). For each class, the fluorescence image (left) and merged fluorescence-bright-field image (right) are shown. (c) An *R. pseudoacacia* pollen tube stained with FM4-64 after growth at 28 °C, showing intact plasma membranes. Left, fluorescence image; right, merged fluorescence-bright-field image. (d) *R. pseudoacacia* pollen tubes after the same heat-shock treatment as in (b): 95.7% retained an intact plasma membrane (left), whereas 4.3% showed small vesicles budding from the plasma membrane (right, yellow arrows) (*n* = 62 pollen tubes). Scale bars, 10 μm (a)−(d). (e) F-actin organization in *A. thaliana* and *R. pseudoacacia* pollen tubes at 28 °C and after heat shock (28 °C for 3 h in the dark, then 37 °C for 30 min), visualized with Actin-Tracker. Yellow dashed boxes mark the subapical region used for quantification. Scale bar, 10 μm. (f) F-actin fluorescence intensity in the subapical region shown in (e). Data are mean ± SEM (*n* = 13, 17, 14, and 15 pollen tubes for *A. thaliana* 28 °C, *A. thaliana* 37 °C, *R. pseudoacacia* 28 °C, and *R. pseudoacacia* 37 °C, respectively); each dot represents an individual pollen tube. Two-tailed Student's *t*-test, ** *p* < 0.01; N.S., not significant.

Given the established link between actin cytoskeleton integrity and thermotolerance in pollen tubes^[39,40]^, we next examined F-actin organization using an F-actin tracker. At 28 °C, *A. thaliana* pollen tubes showed dense F-actin accumulation in the subapical region (Fig. 3e). Upon heat shock at 37 °C, F-actin levels in this region declined significantly, with a concomitant decrease in overall cytoskeletal density (Fig. 3e, f). By contrast, *R. pseudoacacia* pollen tubes maintained stable F-actin abundance and distribution in the subapical region at 37 °C, with no significant difference from 28 °C controls (Fig. 3e, f). This contrast suggests that F-actin stability in *R. pseudoacacia* may support sustained vesicle trafficking and membrane repair, thereby contributing to plasma membrane integrity under high temperatures.

### RpANX2 regulates pollen tube growth in *R. pseudoacacia*

ANXUR (ANX) proteins are essential for pollen tube growth and integrity across multiple angiosperms^[41−44]^. To investigate whether this pathway is conserved in *R. pseudoacacia*, we identified putative orthologs of *A. thaliana* ANX. BLAST analysis revealed that RpANX2 shares high sequence and structural similarity with *A. thaliana* ANX2 (Supplementary Fig. S1a, S1b). RT-PCR confirmed *RpANX2* expression in *R. pseudoacacia* pollen (Fig. 4a).

**Figure 4:**
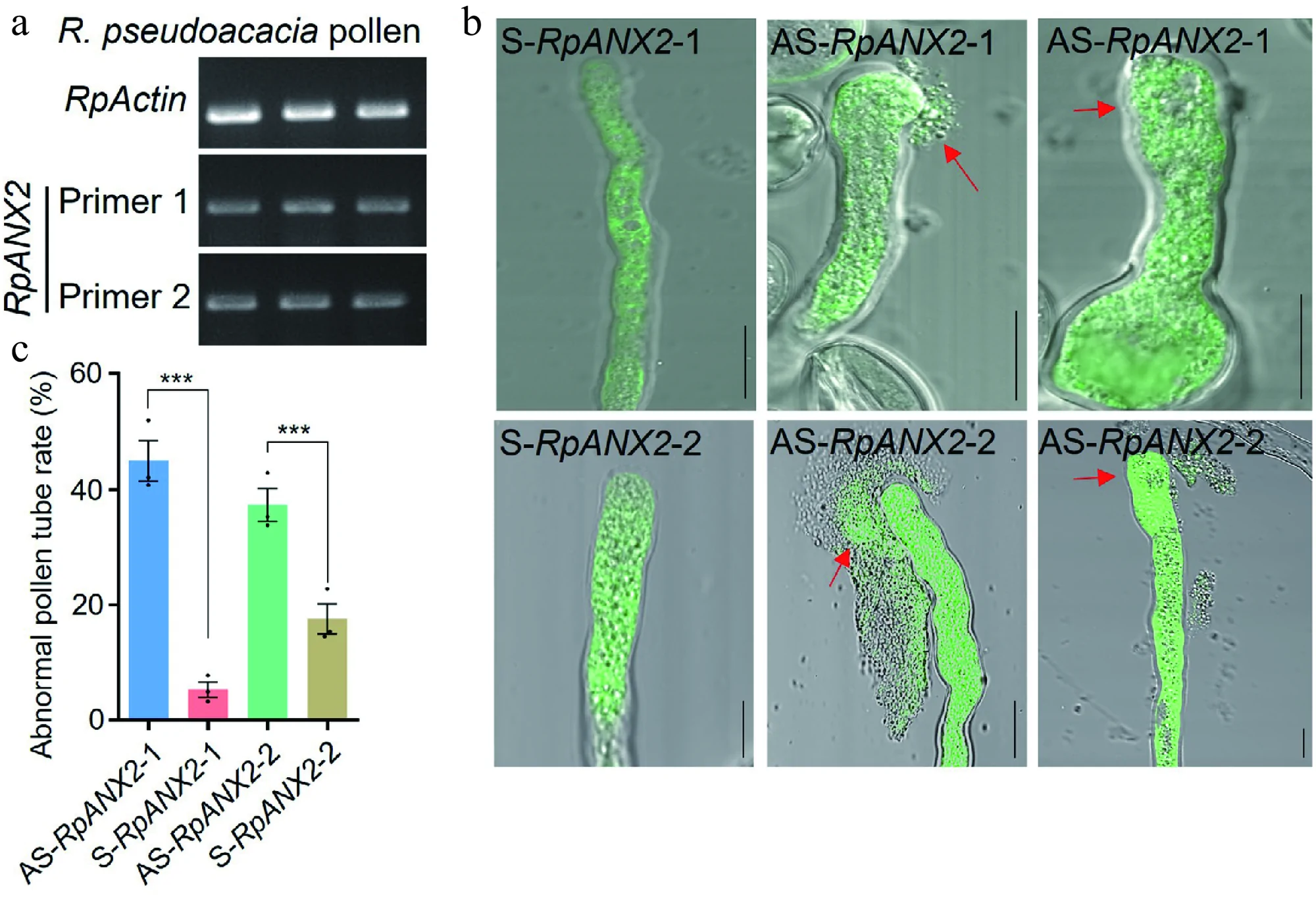
RpANX2 regulates *R.* *pseudoacacia* pollen tube growth. (a) Reverse transcription-polymerase chain reaction (RT-PCR) analysis of *RpANX2* expression in *R. pseudoacacia* pollen, performed with two independent primer pairs (primer 1 and primer 2). *RpActin* served as an internal reference gene. (b) Representative images of pollen tubes treated with antisense oligonucleotides targeting *RpANX2* (AS-*RpANX2*-1 and AS-*RpANX2*-2) or with the corresponding sense-strand control oligonucleotides (S-*RpANX2*-1 and S-*RpANX2*-2). Two representative abnormal phenotypes are shown for each antisense treatment; red arrows indicate sites of tube rupture or swelling. Scale bars, 10 μm. (c) Percentage of abnormal pollen tubes for the treatments shown in (b). Data are mean ± SEM (*n* = 3 biological replicates, ≥ 60 pollen tubes per replicate); each dot represents one biological replicate. Two-tailed Student's *t*-test; *** *p* < 0.001.

To assess *RpANX2* function, we used antisense oligodeoxynucleotides (AS-ODNs) to suppress its expression^[41]^. Fluorophore-labeled AS-*RpANX2* was delivered into pollen tubes, causing severe morphological defects including rupture and apical bulging (Fig. 4b). In contrast, sense controls (S-*RpANX2*) showed minimal abnormalities (Fig. 4b, c). Quantification confirmed that AS-*RpANX2* treatment significantly increased the proportion of abnormal pollen tubes (Fig. 4c). Confocal imaging showed that both AS-*RpANX2* and S-*RpANX2* localized to the cytoplasm, excluding delivery artifacts (Fig. 4b). In *A. thaliana*, ANX proteins interact with Rapid Alkalinization Factor (RALF) peptides to regulate pollen tube growth. We identified *R. pseudoacacia* *RALF* homologs (*RpRALF*s) with high sequence similarity to *A. thaliana* *RALF4/19* (Supplementary Fig. S1c). These findings suggest that the RALF-ANX signaling module is conserved in *R. pseudoacacia*.

### Aberrant pollen tube guidance limits seed set in *R. pseudoacacia*

*R. pseudoacacia* exhibits a markedly low seed set (~25%), in stark contrast to the near-complete seed set of *A. thaliana* (~95%; Fig. 5a, d; Supplementary Fig. S2). We therefore tracked pollen tube behavior following pollination. *R. pseudoacacia* pollen germinated normally on the stigma and grew through the style (Fig. 5b, c); however, only ~45% of ovules were successfully targeted by pollen tubes, a proportion significantly lower than that in *A. thaliana* (Fig. 5c, d). These observations suggest that defective pollen tube guidance, and possibly compromised seed development, underlie the low seed set.

**Figure 5:**
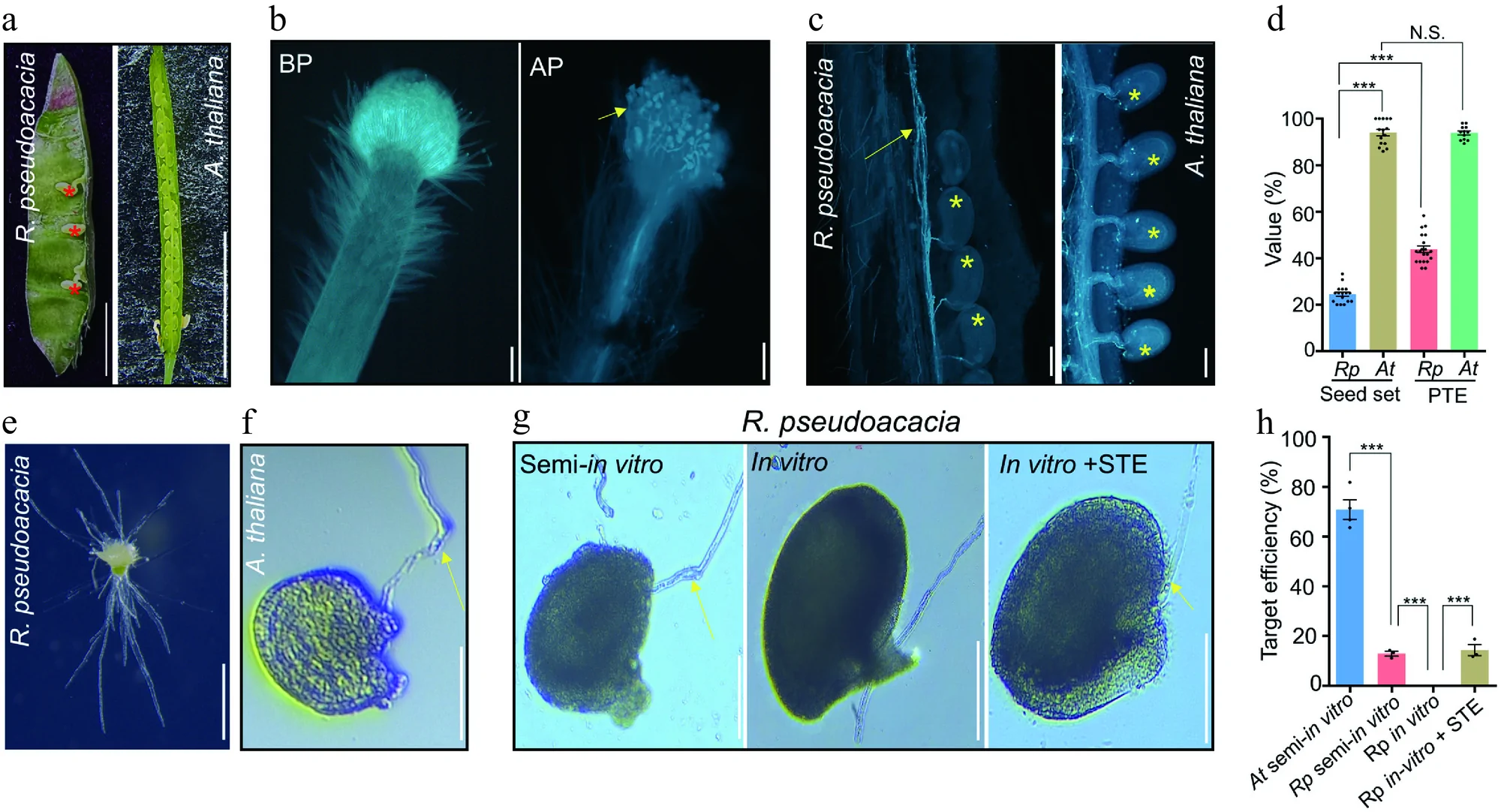
Aberrant pollen tube guidance limits seed set in *R. pseudoacacia*. (a) Seed production in fruits of *R. pseudoacacia* (legume pod, left) and *A. thaliana* (silique, right). Red asterisks indicate developed seeds in the *R. pseudoacacia* pod; the majority of ovules aborted. Scale bars, 1 cm. (b) Aniline blue staining of an *R. pseudoacacia* stigma before pollination (BP, left) and after pollination (AP, right). The yellow arrowhead indicates pollen grains germinating on the stigma. Scale bars: 50 μm. (c) Aniline blue staining of *R. pseudoacacia* (left) and *A. thaliana* (right) pistils after pollination, showing pollen tube growth through the style and ovule targeting. Yellow arrow, pollen tubes; yellow asterisks, ovules reached by a pollen tube. Scale bars, 100 μm. (d) Quantification of seed set shown in (a) and pollen tube targeting efficiency (PTE, the percentage of ovules reached by at least one pollen tube) shown in (c) for both species. Data are mean ± SEM (*n* = 195, 571, 274, and 369 ovules for *R. pseudoacacia* seed set, *A. thaliana* seed set, *R. pseudoacacia* PTE, and *A. thaliana* PTE, respectively); each dot represents one biological replicate. Two-tailed Student's *t*-test; *** *p* < 0.001; N.S., not significant. *Rp*, *R. pseudoacacia*; *At*, *A. thaliana*. (e) Semi-*in vitro* pollen tube guidance assays. *R. pseudoacacia* pollen was germinated on an excised stigma, and pollen tubes emerging from the cut end of the style were used for subsequent ovule attraction assays. Scale bar, 100 μm. (f) An *A. thaliana* ovule attracting a conspecific pollen tube (yellow arrow) in the semi-*in vitro* assay. Scale bar, 100 μm. (g) Attraction of *R. pseudoacacia* pollen tubes to unfertilized *R. pseudoacacia* ovules: a semi-*in vitro-*grown pollen tube (left), an *in vitro*-grown pollen tube (middle), and an *in vitro*-grown pollen tube supplemented with stigma exudates (STE) (right). Yellow arrows indicate pollen tubes growing toward the ovule micropyle. Scale bars, 100 μm. (h) Quantification of ovule targeting efficiency for the assays shown in (f) and (g). Data are mean ± SEM (*n* = 18, 25, 19, and 27 ovules for *A. thaliana* pollen tubes and *R. pseudoacacia* pollen tubes grown semi-*in vitro*, *in vitro,* and *in vitro* with STE, respectively); each dot represents one biological replicate. Two-tailed Student's *t*-test; *** *p* < 0.001.

To quantify the efficiency of ovule attraction, we established a semi-*in vitro* pollen tube guidance assay (Fig. 5e). Whereas *A. thaliana* ovules efficiently attracted conspecific pollen tubes (Fig. 5f), unfertilized *R. pseudoacacia* ovules attracted only ~13% of semi-*in vitro* germinated pollen tubes, but almost none of *in vitro *germinated pollen tube (Fig. 5g, h). Notably, supplementing the assay with stigma exudates (STE) restored ovule attraction (Fig. 5g, h), indicating that stigma-derived signals are required to guide *R. pseudoacacia* pollen tubes to the ovule. These results indicate that an intrinsic defect in ovule attraction, together with a strict dependence on stigma-derived guidance signals, limits reproductive success in *R. pseudoacacia*.

### *R. pseudoacacia* pollen tubes overcome intergeneric pre-fertilization barriers

To assess the hybridization potential of *R. pseudoacacia*, we pollinated the stigmas of *A. thaliana*, a distantly related Brassicaceae species, with *R. pseudoacacia* pollen. Despite this wide phylogenetic distance, *R. pseudoacacia* pollen germinated on *A. thaliana* stigmas and penetrated the style (Fig. 6a). At 24 hap, 3.6% of *A. thaliana* ovules (*n *= 424) had been targeted by *R. pseudoacacia* pollen tubes, and the targeted ovules exhibited swelling around the micropyle (Fig. 6a). Nevertheless, no seeds were obtained (Supplementary Fig. S3a), indicating post-zygotic incompatibility.

**Figure 6:**
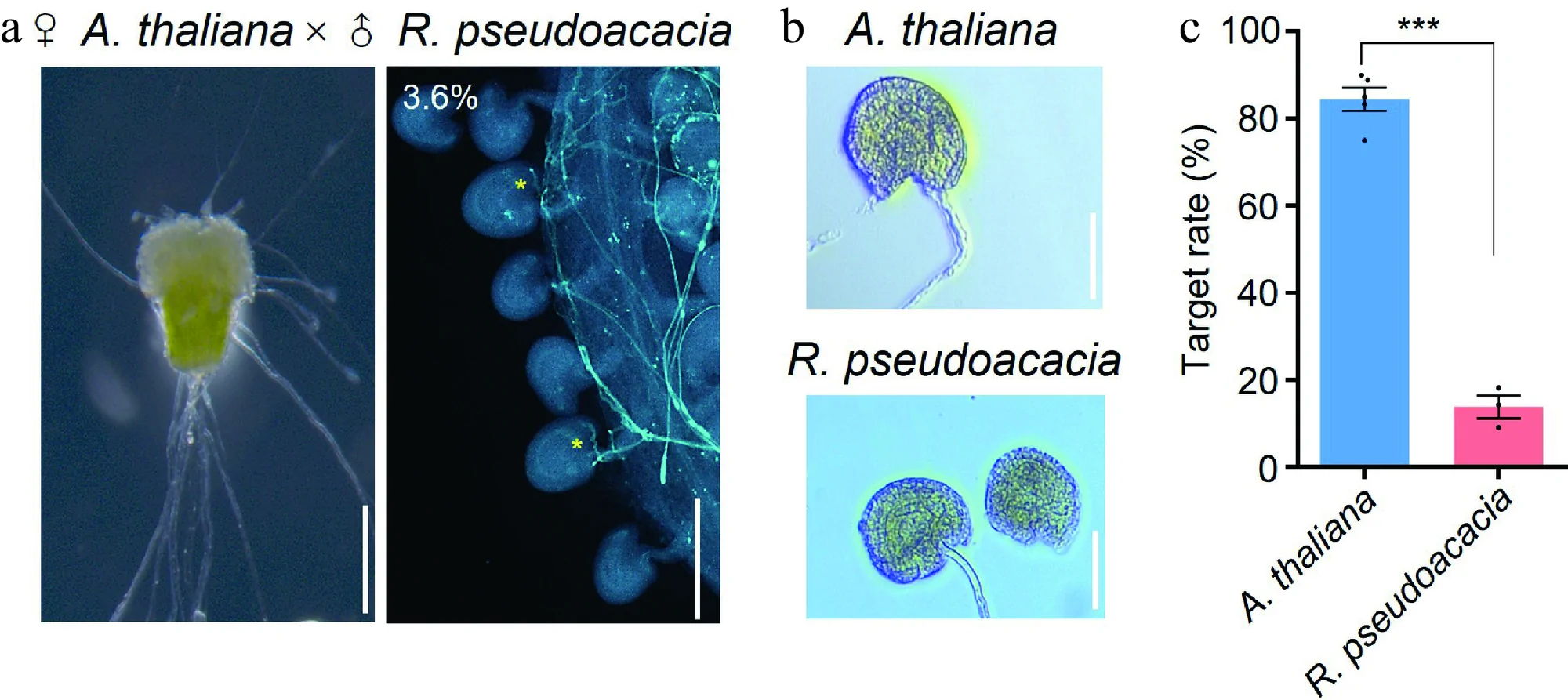
*R. pseudoacacia* pollen tubes attracted by *A. thaliana* ovules. (a) Cross-pollination of *A. thaliana* (♀) with *R. pseudoacacia* pollen (♂). Left, semi-*in vitro* assay showing *R. pseudoacacia* pollen germinated on an *A. thaliana* stigma, with pollen tubes emerging from the cut end of the style; right, an aniline blue-stained *A. thaliana* pistil at 24 h after pollination, showing *R. pseudoacacia* pollen tubes that grew through the style and reached ovules (yellow asterisks; 3.6% of 424 ovules examined). Scale bars, 200 μm. (b) Ovule attraction assays in which *A. thaliana* ovules were co-cultured with semi-*in vitro*-grown pollen tubes of *A. thaliana* (upper panel) or *R. pseudoacacia* (lower panel). Scale bars, 100 μm. (c) Quantification of ovule targeting rates for the assays shown in (b). Data are mean ± SEM (*n* = 28 and 36 ovules for *A. thaliana* and *R. pseudoacacia* pollen tubes, respectively); each dot represents one biological replicate. Two-tailed Student's *t*-test; * *p* < 0.05.

Semi*-in vitro* ovule attraction assays confirmed that *A. thaliana* ovules attract *R. pseudoacacia* pollen tubes, although the efficiency was significantly lower than that of *A. thaliana* pollen tubes (Fig. 6b, c). This cross-species attraction implies that *R. pseudoacacia* pollen tubes possess receptors capable of perceiving ovule-derived attractants from *Arabidopsis*. In *A. thaliana*, POLLEN-SPECIFIC RECEPTOR-LIKE KINASE 6 (PRK6) is a pollen tube receptor that directly perceives the ovule-derived attractant AtLURE1^[45,46]^; we therefore investigated its *R. pseudoacacia* homologs as candidates mediating this cross-species response. Phylogenetic analysis identified three PRK6 homologs in the *R. pseudoacacia* genome (RpPRK6a, RpPRK6b, and RpPRK6c; Supplementary Fig. S3b), of which RpPRK6b is expressed in pollen (Supplementary Fig. S3c). Sequence alignment showed that the RpPRK6 proteins share high similarity with AtPRK6 in the intracellular kinase domain but diverge substantially in the extracellular leucine-rich repeat (LRR) domain (Supplementary Fig. S3d). Consistently, structural superposition showed that the intracellular kinase domains of RpPRK6b and AtPRK6 adopt a highly conserved fold (RMSD = 0.519 Å; Supplementary Fig. S4). In line with this divergence in the predicted ligand-binding region, *R. pseudoacacia* pollen tubes showed no detectable response to AtLURE1.2, the cognate ligand of AtPRK6, whereas *A. thaliana* pollen tubes responded strongly (Supplementary Fig. S5). Moreover, no AtLURE1.2 homologs could be identified in the *R. pseudoacacia* genome, suggesting that an as-yet-unknown attractant mediates this cross-species guidance. Collectively, these findings demonstrate that *R. pseudoacacia* pollen tubes can overcome intergeneric pre-fertilization barriers and respond to heterospecific ovule signals via a LURE1-independent mechanism, providing a theoretical basis for distant hybridization breeding in leguminous trees.

## Discussion

Perennial plants exhibit unique reproductive traits, such as repeated vegetative-reproductive cycles, diverse gametophytic patterns, and the potential for apomixis or intergeneric hybridization. These traits cannot be fully elucidated using annual model species such as *Arabidopsis*^[14,20]^. To help close the longstanding gap in perennial reproductive research^[20]^, this study systematically characterized the reproductive process of *R. pseudoacacia*, from pollen germination to double fertilization, and established standardized experimental protocols for this species. These results provide the first integrated description of fertilization in a perennial legume tree. They also reveal several features that warrant further mechanistic investigation, including reproductive thermotolerance, aberrant pollen tube guidance, and intergeneric pollen tube compatibility.

Previous studies on *R. pseudoacacia* reproduction have focused narrowly on seed set^[47−49]^, with limited tools available for mechanistic investigation. Here, we addressed this limitation by developing two critical resources. First, we established a species-specific pollen germination medium that yields over 90% germination (Fig. 1c, d), a marked improvement over previously reported low rates^[36]^. Second, we developed a transient gene suppression system using AS-ODNs to target reproductive genes such as *RpANX2* (Fig. 4b, c). These tools, combined with fluorescent probes established in *A. thaliana* (e.g., DAF-DA, H2DCFDA, and Actin-Tracker) for visualizing NO, ROS, and actin filaments (Fig. 1e), provide a flexible experimental toolkit for comparative and molecular studies of perennial pollen biology.

Among existing woody study species, *Populus* has the most extensively developed genetic toolkit, including established *Agrobacterium*-mediated transformation, genome editing systems, and rich genomic resources^[22]^. For reproductive studies, however, *Populus* presents several constraints, including a prolonged juvenile phase, catkin-based reproductive structures that are difficult to manipulate, and a narrow flowering window. In contrast, *R. pseudoacacia* flowers within a few years after germination and produces abundant flowers that are readily amenable to experimental manipulation. It also exhibits reproductive traits not reported in *Populus*, namely pollen germination and tube growth at temperatures lethal to *A. thaliana* pollen (Fig. 2c, d) and pollen tube growth across intergeneric pre-fertilization barriers (Fig. 6a). The two species therefore have complementary features. *Populus* is currently more amenable to genetic manipulation, whereas *R. pseudoacacia* offers distinct advantages for studies of reproductive physiology, pollen thermotolerance, and distant hybridization.

Beyond *Populus*, other perennials have contributed to the study of specific reproductive questions. *Citrus* species have been instrumental in elucidating apomixis^[16,17]^, *Malus* and *Pyrus* in interspecific and intergeneric hybridization^[18,19]^, *Actinidia* (kiwifruit) in dioecy and sex determination^[50]^, and *Prunus* species in gametophytic self-incompatibility^[51]^. Each of these species, however, addresses a specialized question and carries its own practical constraints. By contrast, *R. pseudoacacia* combines several advantageous attributes within a single species, including accessible reproductive organs, a relatively short juvenile phase, an extended flowering period, reproductive thermotolerance, and the capacity for distant hybridization. It therefore represents a useful addition to the perennials available for reproductive research.

A key finding of this study is the high reproductive thermotolerance of *R. pseudoacacia*. Whereas *A. thaliana* pollen fails to germinate at 37 °C, *R. pseudoacacia* pollen maintains over 20% germination at this temperature and exceeds 70% across 25−34 °C (Fig. 2c, d), a trait that may contribute to its ability to colonize diverse climatic zones, from temperate to semi-arid regions^[25]^. This thermotolerance was associated with two cellular features, namely plasma membrane integrity and F-actin cytoskeleton stability. At 37 °C, 95.7% of *R. pseudoacacia* pollen tubes retained intact plasma membranes, compared with only 21.2% in *A. thaliana* (Fig. 3b, d). F-actin density and subapical localization were maintained under heat stress, whereas *A. thaliana* pollen tubes exhibited pronounced F-actin disassembly (Fig. 3e, f). These observations are consistent with previous work linking F-actin stability to pollen thermotolerance^[5,39,40]^ and the plasma membrane to thermal sensing and acquired thermotolerance^[52,53]^, and extend these associations to pollen of a perennial tree species. The underlying molecular basis remains to be determined, and two hypotheses are worth testing. Actin-binding proteins in *R. pseudoacacia* may exhibit greater heat stability than their *A. thaliana* orthologs^[39,40]^. Its pollen tubes may possess enhanced ROS scavenging or signaling regulation, as heat-induced ROS bursts that disrupt pollen tube membranes in annuals^[5,54]^ caused little such damage in *R. pseudoacacia*. Testing these hypotheses could reveal divergent heat-adaptation strategies between annuals and perennials.

We recognize that our conclusions rest largely on descriptive analyses, which reveal correlations rather than causal mechanisms. The AS-ODN-mediated suppression of *RpANX2*, which caused severe pollen tube defects (Fig. 4b, c), provides an initial perturbation-based validation, but definitive causal analysis will require stable genetic transformation in *R. pseudoacacia*. We therefore present these findings as a cytological and methodological foundation for future functional studies.

Our results also provide insight into the low seed set of *R. pseudoacacia* (Fig. 5a), previously described as selective abortion^[48,55,56]^. Aberrant pollen tube guidance emerges as a key limiting factor. Only ~45% of ovules were targeted by pollen tubes (Fig. 5c, d), and unfertilized ovules attracted almost none of *in vitro*-grown pollen tubes, a defect partially rescued by stigma exudates (Fig. 5g, h). Fertilization is thus constrained by a dual barrier, namely intrinsic defects in ovule-derived attraction and a dependence on stigma signals that activate pollen tube responsiveness. These findings bear on the evolution of selective abortion in perennials^[56,57]^, which may reflect resource reallocation toward high-quality seeds^[55]^, purging of deleterious mutations^[57]^, or both. Notably, the seed set (~25%) falls well below the ovule targeting rate (~45%), indicating that a substantial fraction of targeted ovules fails to develop into seeds. This pattern is more consistent with the genetic load hypothesis, although confirmation will require transcriptomic comparison of aborting and viable ovules.

Notably, *R. pseudoacacia* pollen was able to overcome intergeneric reproductive barriers. Pollen of this legume tree (Fabaceae) germinated on *A. thaliana* (Brassicaceae) stigmas, penetrated the style, and targeted 3.6% of ovules (Fig. 6a), although post-zygotic incompatibility prevented seed formation (Supplementary Fig. S3a). Such pre-zygotic compatibility is rarely observed in crosses between distantly related species and is not fully accounted for by traditional models of reproductive isolation. Mechanistically, it may involve RpPRK6, the *R. pseudoacacia* homolog of the receptor AtPRK6, which shares high similarity with AtPRK6 in the intracellular signaling domain but diverges in the extracellular ligand-binding LRR domain (Supplementary Fig. S3b−S3d). This divergence likely explains why *R. pseudoacacia* pollen tubes did not respond to AtLURE1.2 (Supplementary Fig. S5) yet could still be guided by *A. thaliana* ovules, and it raises the hypothesis that *A. thaliana* ovules produce alternative attractants perceived by RpPRK6. This hypothesis could be tested by identifying ovule-secreted RpPRK6-binding proteins. The proposed role of RpPRK6, however, currently rests on sequence and structural comparisons and ligand-response assays, and direct functional evidence, such as receptor-ligand binding or domain-swap experiments, will be required to validate it. These findings could also inform strategies to overcome pre-zygotic incompatibility in interspecific legume crosses.

Beyond these molecular considerations, *R. pseudoacacia* also presents practical limitations for reproductive manipulation. Its flowers are small (corolla approximately 1.6 cm long) and hermaphroditic, requiring careful and complete emasculation before controlled crosses. In practice, this can be achieved by removing undehisced anthers before anthesis under a dissecting microscope, after which the inflorescence is bagged to prevent unwanted pollination. These floral features thus add experimental complexity but do not fundamentally limit the tractability of this species. Apart from these floral traits, two further limitations remain. First, although the genome of *R. pseudoacacia* has been sequenced^[58]^, a robust genetic transformation system is still lacking, hindering stable gene knockout and overexpression studies. Second, its natural one-month flowering period restricts experimental windows, although this can be extended to several months through cold storage of pollen^[59,60]^ and the use of populations differing in flowering time across regions^[61,62]^.

## Conclusions

This study characterized sexual reproduction in the perennial tree *R. pseudoacacia* and established supporting methods, including optimized cytological assays and antisense oligodeoxynucleotide-mediated gene suppression in pollen. *R. pseudoacacia* pollen tolerated temperatures lethal to *A. thaliana* pollen, a thermotolerance linked to plasma membrane stability and F-actin organization. The conserved ANX-RALF module component RpANX2 was required for normal tube growth. Seed set appears limited mainly at the guidance step, since unfertilized ovules barely attracted tubes *in vitro* unless stigma exudates were supplied. Yet the pollen crossed pre-fertilization barriers on *A. thaliana* pistils, a partial compatibility that may be explained by divergent RpPRK6. These results provide a framework for reproductive studies in woody perennials and identify pollen tube guidance as a breeding target.

## SUPPLEMENTARY DATA

Supplementary data to this article can be found online.

## Data Availability

The data supporting the findings of this study are available within the article and its supplementary information.
