# Supplementary material for: Characterization of the sexual reproductive process in the perennial tree Robinia pseudoacacia
Source: For Res (Fayettev). 2026 Sep 8;6:e027. doi: 10.48130/forres-0026-0027 (PMC13662927; doi:10.48130/forres-0026-0027)
Supplement: Supplementary file 1 [file forres-2026-6-1-0027-S1.zip › 10.48130_forres-0026-0027-Suppl-FigureS2.pdf]

Supplemental figure 2

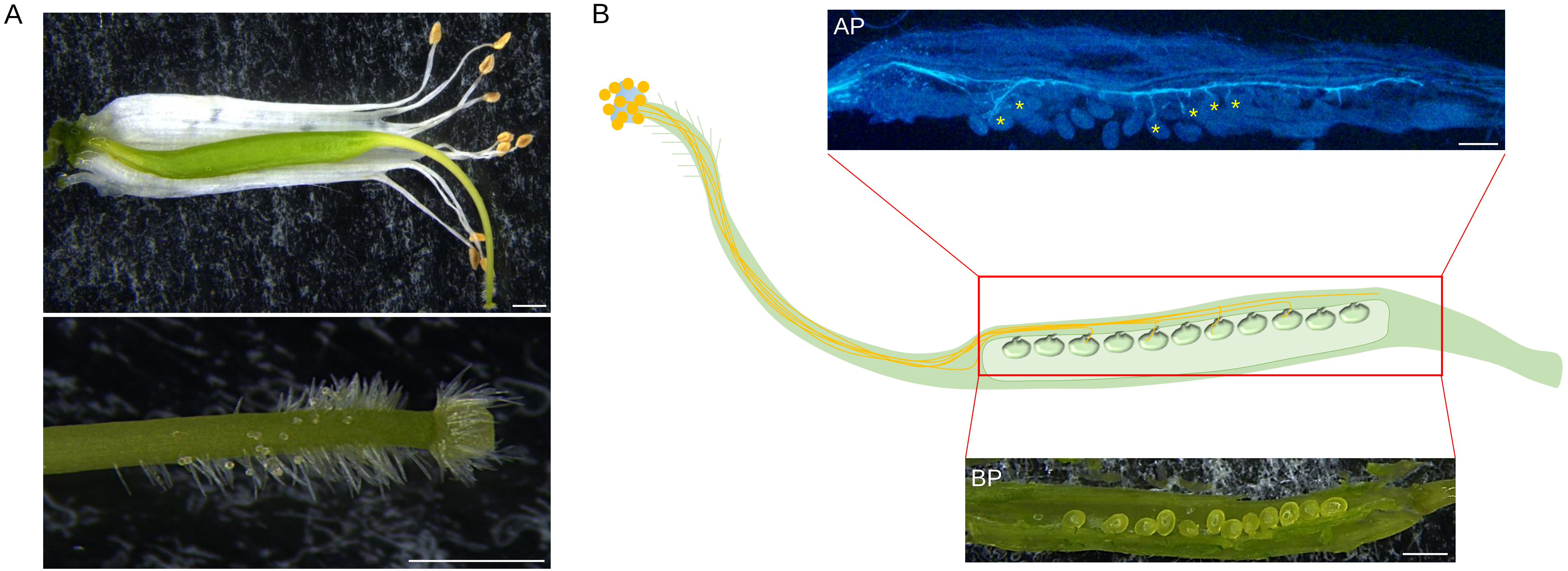

Fig. S2 Structural anatomy of the *R. pseudoacacia* pistil and pollen tube growth in vivo.

(A) A dissected R. pseudoacacia flower showing the pistil (upper panel), and a close-up view of the stigma and style covered with stigmatic papillae (lower panel). Scale bars, 500 µm.

(B) Upper panel: an aniline blue-stained *R. pseudoacacia* pistil after pollination (AP), showing pollen tubes growing through the style and targeting ovules (yellow asterisks); lower panel: a dissected ovary before pollination (BP), showing the longitudinal arrangement of ovules. Scale bars, 200 µm. BP, before pollination; AP, after pollination.
