# Supplementary material for: Characterization of the sexual reproductive process in the perennial tree Robinia pseudoacacia
Source: For Res (Fayettev). 2026 Sep 8;6:e027. doi: 10.48130/forres-0026-0027 (PMC13662927; doi:10.48130/forres-0026-0027)
Supplement: Supplementary file 1 [file forres-2026-6-1-0027-S1.zip › 10.48130_forres-0026-0027-Suppl-FigureS3.pdf]

Supplemental figure 3

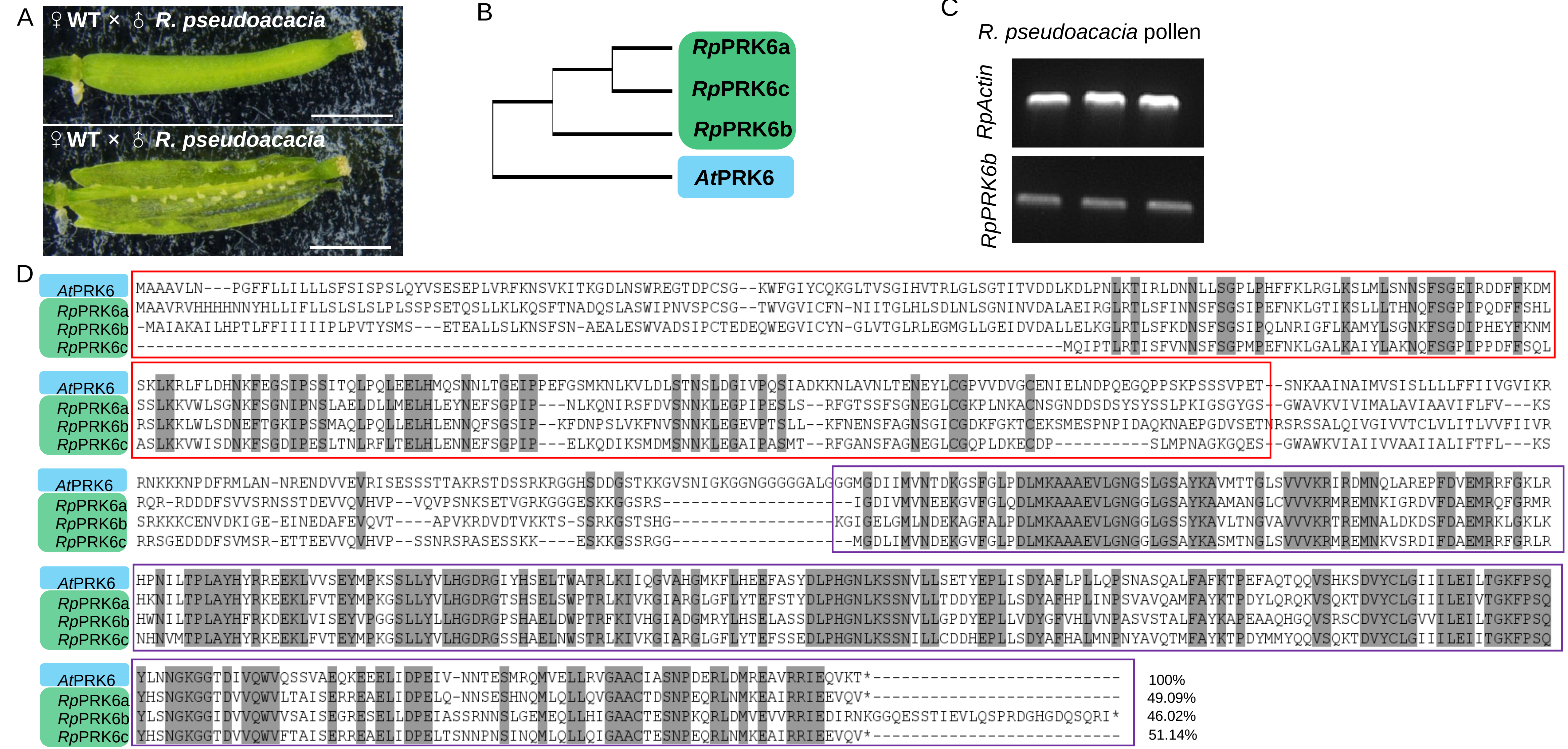

Fig. S3 PRK6 homologs in *R. pseudoacacia* and their potential involvement in cross-species pollination.
(A) Mature siliques of wild-type *A. thaliana* (♀) pollinated with *R. pseudoacacia* pollen (♂), showing the absence of seed development. Scale bars, 1 mm.
(B) Phylogenetic analysis of PRK6 homologs from *A. thaliana* (AtPRK6) and *R. pseudoacacia* (RpPRK6a, RpPRK6b, and RpPRK6c).
(C) Reverse transcription-polymerase chain reaction (RT-PCR) analysis of *RpPRK6b* expression in *R. pseudoacacia* pollen; *RpActin* served as the internal reference gene.
(D) Amino acid sequence alignment of PRK6 homologs from *A. thaliana* and *R. pseudoacacia*. Gray shading indicates identical residues; the red box marks the extracellular leucine-rich repeat (LRR) domain, and the purple box marks the intracellular kinase domain. Numbers on the right indicate the percentage identity of each sequence to AtPRK6 (100%, 49.09%, 46.02%, and 51.14% for AtPRK6, RpPRK6a, RpPRK6b, and RpPRK6c, respectively).
