# Supplementary material for: Characterization of the sexual reproductive process in the perennial tree Robinia pseudoacacia
Source: For Res (Fayettev). 2026 Sep 8;6:e027. doi: 10.48130/forres-0026-0027 (PMC13662927; doi:10.48130/forres-0026-0027)
Supplement: Supplementary file 1 [file forres-2026-6-1-0027-S1.zip › 10.48130_forres-0026-0027-Suppl-FigureS4.pdf]

Supplemental figure 4

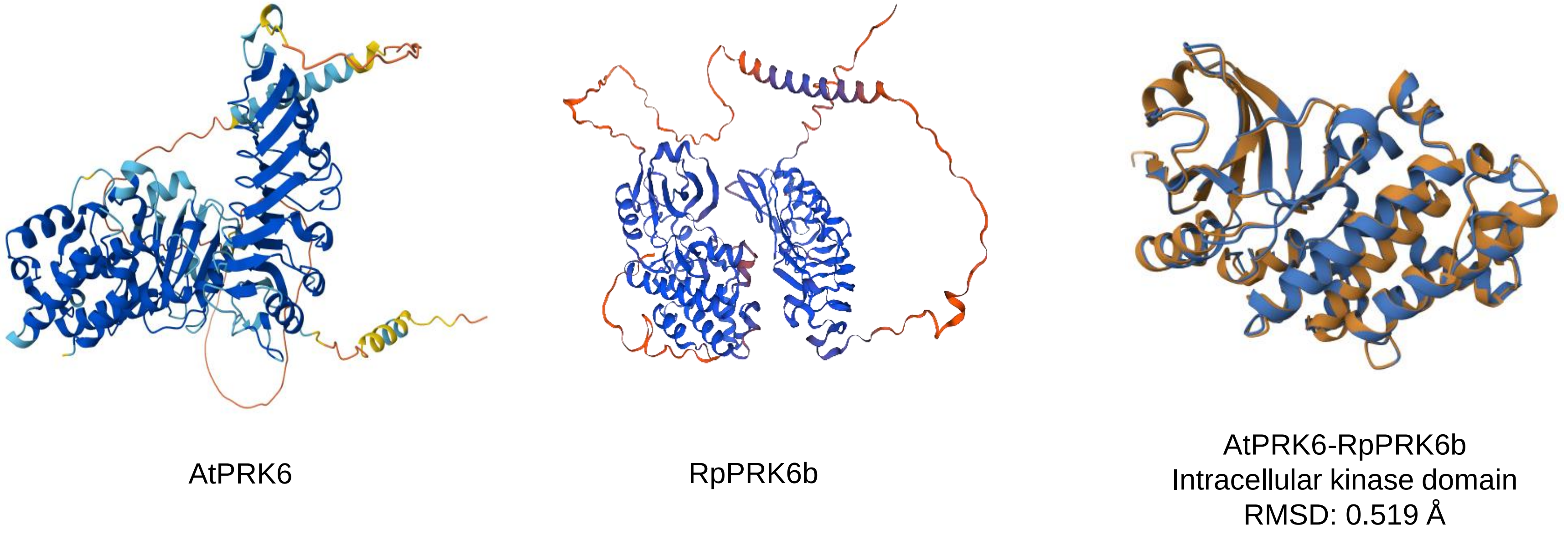

Fig. S4 Predicted structures of PRK6 homologs from *A. thaliana* and *R. pseudoacacia*.

Predicted three-dimensional structures of AtPRK6 (left) and RpPRK6b (middle), and structural superposition of the intracellular kinase domains of AtPRK6 and RpPRK6b (right). Root mean square deviation (RMSD) = 0.519 Å. Structures were predicted with AlphaFold2. Single structures are colored by AlphaFold2 per-residue confidence (left and middle). AtPRK6 is shown in blue and RpPRK6b in brown in the superposition (right).
