# Supplementary material for: Characterization of the sexual reproductive process in the perennial tree Robinia pseudoacacia
Source: For Res (Fayettev). 2026 Sep 8;6:e027. doi: 10.48130/forres-0026-0027 (PMC13662927; doi:10.48130/forres-0026-0027)
Supplement: Supplementary file 1 [file forres-2026-6-1-0027-S1.zip › 10.48130_forres-0026-0027-Suppl-FigureS5.pdf]

Supplemental figure 5

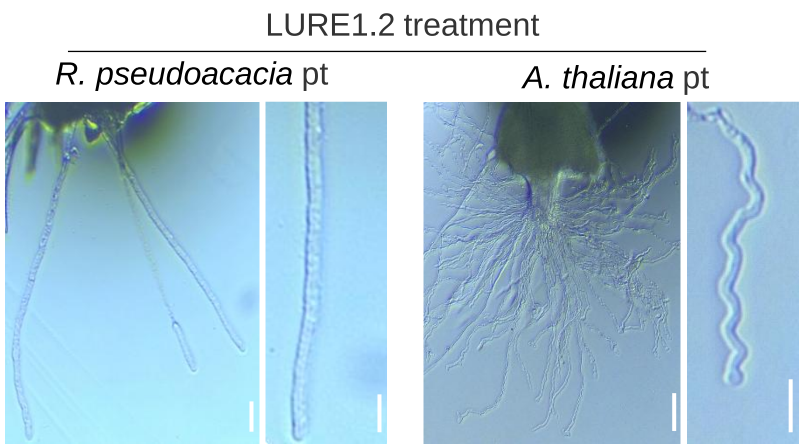

Fig. S5 *R. pseudoacacia* pollen tubes do not respond to AtLURE1.2.

Growth responses of *R. pseudoacacia* (left) and *A. thaliana* (right) pollen tubes (pt) after 1 h of treatment with 1 µM AtLURE1.2 uniformly applied in the medium. *R. pseudoacacia* pollen tubes showed no detectable change in growth direction, whereas *A. thaliana* pollen tubes exhibited marked changes in growth orientation. Scale bars, 10 µm.
