# Supplementary material for: Characterization of the sexual reproductive process in the perennial tree Robinia pseudoacacia
Source: For Res (Fayettev). 2026 Sep 8;6:e027. doi: 10.48130/forres-0026-0027 (PMC13662927; doi:10.48130/forres-0026-0027)
Supplement: Supplementary file 1 [file forres-2026-6-1-0027-S1.zip › 10.48130_forres-0026-0027-Suppl-TableS1.pdf]

Table S1. Oligonucleotides used in this study.
Primer sequences for reverse transcription-polymerase chain reaction (RT-PCR), and antisense (AS-) and sense (S-) oligodeoxynucleotides (ODNs) targeting RpANX2. All sequences are given in the 5’-to-3’ direction; lowercase letters indicate phosphorothioate-modified nucleotides.

Name	Sequences (5'-3')
AS-ANX2-1	aacgTTGTTGCCGCTCcatgC
S-ANX2-1	gcatGGAGCGGCAACAacgtT
AS-ANX2-2	gctgCCATGTCATTGTtattG
S-ANX2-2	caatAACAATGACATGgcagC
rt-ANX2-1F	AATGCCCTTCCTGCATGGA
rt-ANX2-1R	CGCTTATCAGGTTGAACAGCG
rt-ANX2-2F	CAACACATCTAGCTGGTTGCC
rt-ANX2-2R	TTGATTGCCACTTTGAACCCAC
rt-PRK6b-F	GGGTGGCTGATTCTATACCTTG
rt-PRK6b-R	GTTGTGGGATGGAACCTGAG
rt-ACT2-F	GAATGGTGAAGGCTGGATTTGC
rt-ACT2-R	CTCAGGAGCAACACGAAGTTC
