# Supplementary material for: Characterization of the sexual reproductive process in the perennial tree Robinia pseudoacacia
Source: For Res (Fayettev). 2026 Sep 8;6:e027. doi: 10.48130/forres-0026-0027 (PMC13662927; doi:10.48130/forres-0026-0027)
Supplement: Supplementary file 1 [file forres-2026-6-1-0027-S1.zip › 10.48130_forres-0026-0027-Suppl-FigureS1.pdf]

# Supplemental figure 1

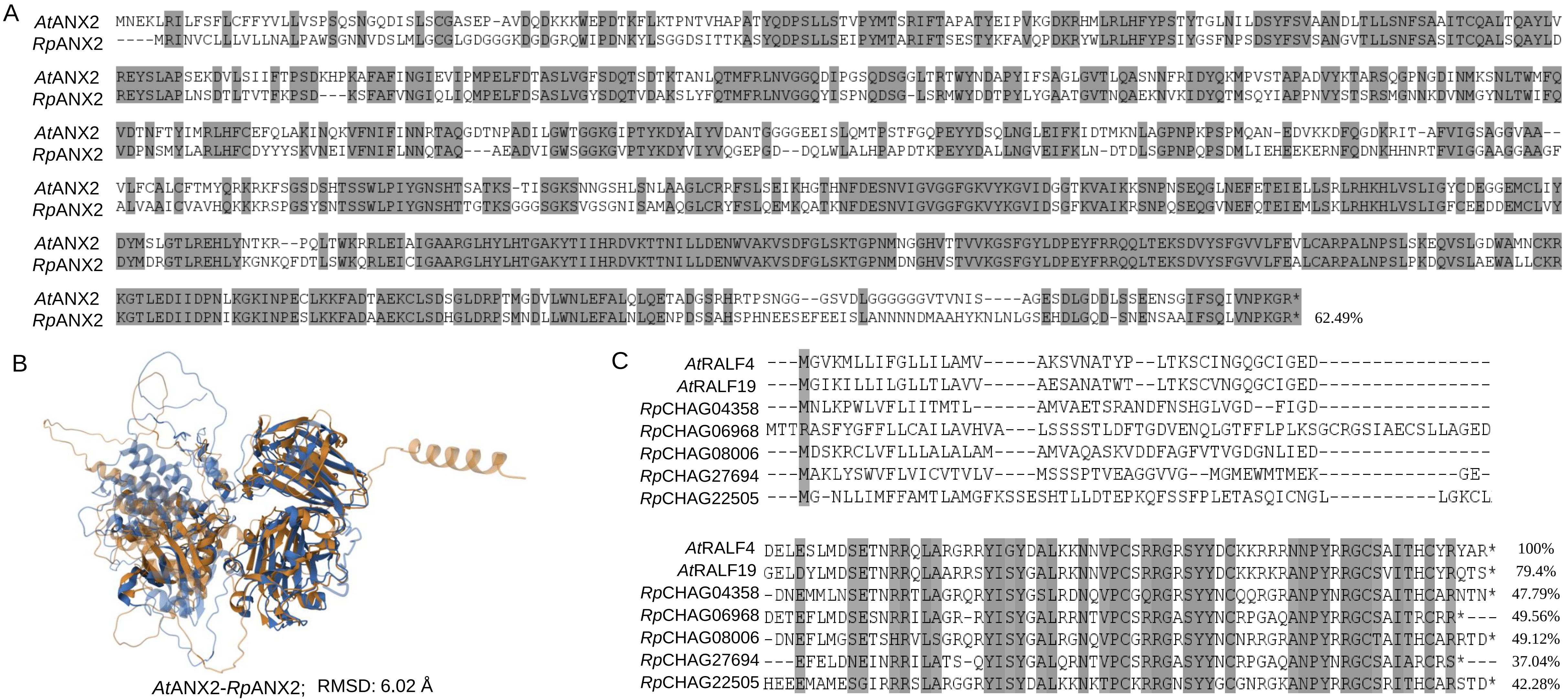


**Fig. S1** Sequence and structural conservation of ANX2 and RALF proteins between *A. thaliana* and *R. pseudoacacia*.

(A) Amino acid sequence alignment of ANX2 from *A. thaliana* (AtANX2) and *R. pseudoacacia* (RpANX2). Gray shading indicates identical residues; the overall sequence identity is 62.49%.

(B) Superposition of the three-dimensional structures of AtANX2 and RpANX2 predicted with AlphaFold2 (root mean square deviation, RMSD = 6.02 Å).

(C) Amino acid sequence alignment of *A. thaliana* RALF4 and RALF19 with five putative *R. pseudoacacia* RALF homologs (RpCHAG04358, RpCHAG06968, RpCHAG08006, RpCHAG22505, and RpCHAG27694). Gray shading indicates identical residues; numbers on the right indicate the percentage identity of each sequence to AtRALF4.
